# Quality of Life in Patients with Malignant Wounds Treated at a Wound Care Clinic

**DOI:** 10.21203/rs.3.rs-4797536/v1

**Published:** 2024-09-02

**Authors:** Anna Chen, Stephen Dusza, Jacqueline Bromberg, Shari Goldfarb, Rachel Sanford, Alina Markova

**Affiliations:** Memorial Sloan Kettering Cancer Center; Memorial Sloan Kettering Cancer Center; Memorial Sloan Kettering Cancer Center; Memorial Sloan Kettering Cancer Center; Memorial Sloan Kettering Cancer Center; Memorial Sloan Kettering Cancer Center

**Keywords:** malignant wounds, cancer care, quality of life, wound care, wound clinic, palliative care

## Abstract

**Background:**

Malignant wounds can present in up to 14.5% of patients with advanced cancer, significantly reducing quality of life (QoL). Management of malignant wounds is generally palliative, with the goal of improving or maintaining QoL. There is a lack of data on the impact of wound care clinics on QoL in patients with malignant wounds.

**Objectives:**

We sought to assess the QoL in patients with malignant wounds attending a wound care clinic. We also aimed to describe the baseline QoL, trends in QoL, physical symptoms, and treatment modalities that affect QoL in patients with malignant wounds over time.

**Methods:**

This retrospective observational study included 36 patients attending a wound care clinic at an oncologic hospital from 1/1/2016–4/1/2023. As part of the standard of care, these patients complete a Skindex-16 QoL survey at each visit. The Skindex-16 is a validated instrument to measure the effects of skin diseases on QoL. Data were extracted from the electronic medical record. Descriptive statistics, graphical methods, and random effects models for change were used to describe the patient population and the QoL measures over time.

**Results:**

Of the 36 patients who completed at least one Skindex-16 questionnaire, 69.4% were female, and 50.0% developed malignant wounds from breast cancer, 30.5% from nonmelanoma skin cancer, and 8.3% from sarcoma. At the initial visit, 86.1% of patients had exudate associated with their malignant wound, 52.7% of patients had malodor, 63.9% had bleeding, 69.4% had pain, and 50% had pruritus. The mean baseline Skindex-16 score was 54.5, falling into the “extremely severe” category, with a mean score of 15.4, 18.8, and 20.3 for the symptoms, emotions, and functioning domains, respectively. Nineteen patients completed at least one additional Skindex-16 questionnaire at follow-up visits (visit two 52.8%, visit three 33.3%, visit four 19.4%, visit five or greater 13.9%). Compared to the mean Skindex-16 score at baseline, there was an 18.5 point improvement at visit 2 (95% CI: 3.3–33.7, p = 0.018).

**Conclusion:**

Malignant wounds severely adversely affect patients’ quality of life. However, patients experienced improved quality of life after being treated at a dedicated wound clinic.

## Introduction

Malignant wounds occur when cancer infiltrates the skin and destroys surrounding vasculature, usually occurring in patients with advanced cancer (1). Malignant wounds can present in up to 14.5% of patients with advanced cancer and are a leading cause of pain and disfigurement, leading to significantly reduced quality of life (QoL) (2, 3). Malignant wounds are characterized by rapid growth with significant physical symptoms including pain, exudate, malodor, bleeding, infection, and necrosis (4). This symptom burden has frequently been associated with psychosocial distress, functional impairment, and severely reduced QoL as shown by Skindex questionnaires, a validated measure of the effects of skin diseases on QoL (5). Studies have shown that patients’ age, pain, wound dressing comfort, bleeding, and malodor have statistically significant negative correlations with QoL (6). Furthermore, psychosocial symptoms of malignant wounds include depression, embarrassment, shame, guilt, stigmatization, and worthlessness (7) which can exacerbate negative QoL. Due to the nature of advanced cancer, management of malignant wounds is generally palliative, with the goal of improving or maintaining QoL.

Several studies have shown the benefit of dedicated wound care clinics on wound healing outcomes and patient quality of life (8–10). Despite evidence for the utility of dedicated wound clinics, there have yet to be studies on the impact of wound care clinics on patients with malignant wounds. We sought to assess the QoL in patients with malignant wounds attending a wound care clinic. We also aimed to describe the baseline QoL, trends in QoL, physical symptoms, and treatment modalities that affect QoL in patients with malignant wounds over time.

## Methods

### Patient population

This single-center retrospective study included 36 patients with malignant wounds seen between 1/1/2016 and 4/1/2023 at a wound care clinic at an oncologic hospital with at least one completed Skindex-16 questionnaire. Patients who did not complete a Skindex-16 questionnaire were excluded from this study. Data was retrieved via a database query using the ICD-10 diagnosis Code C44. 9 and a text search for patients with malignant wounds using the following terms: “malignant wound(s),” “MW,” “malig wound,” “ulcerated tumor,” and “ulcer.” Resulted patients were included based on completion of Skindex-16. Physical symptom (exudate, malodor, bleeding, pruritus) and treatment (dressings, systemic, topical) data were obtained from dermatology provider notes.

### Statistical analysis

Descriptive statistics, graphical methods, and random effects models for change were used to describe the patient population and the QoL measures over time. Data were analyzed using Stata v.16.1, Stata Corporation, College Station, TX.

## Results

### Patient demographics

**Figure 1:**
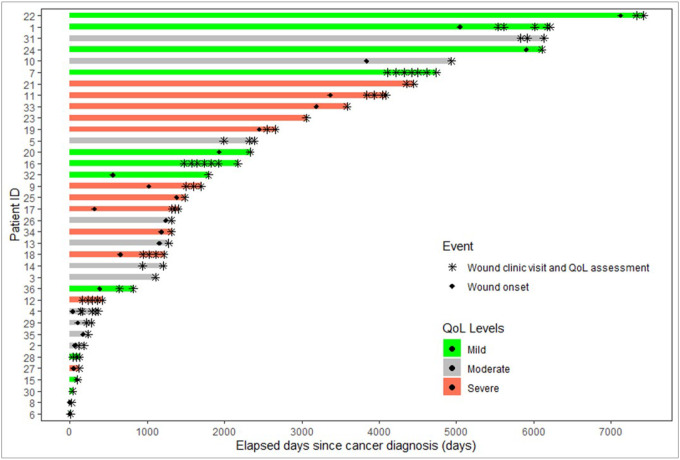
Swimmer plot of all participants from date of cancer diagnosis, including wound onset, wound clinic visit and QoL assessment

Of the 36 patients who completed at least one Skindex-16 questionnaire, 69.4% were female, 63.9% were White, 19.4% were Black, 69% were non-Hispanic, and had an average age of 65 years. Most patients developed malignant wounds from breast cancer (50%), 30.5% from nonmelanoma skin cancer, and 8.3% from sarcoma. Most patients had advanced cancer; 8.3% were stage II, 41.7% stage III, and 50.0% stage IV (Table 1). On average wound clinic patients in this cohort completed 2.3 Skindex-16 surveys over the course of their follow-up (Fig. 1).

### Clinical symptoms and treatment of malignant wounds

At the initial visit, 47.2% of patients had malodor associated with their wound, 63.9% had bleeding, 69.4% had pain, 50.0% had pruritus, and 86.1% of patients had exudate (Table 2). After the initial visit, there was a decrease in the percentage of patients experiencing odor, bleeding, pain, and pruritus at each visit. By visit four, 14.3% of patients had odor, and 28.6% had bleeding, pain, and pruritus. There was not a consistent trend in patients with exudate, which affected most patients at each visit (visit 1 = 86.1%, 2 = 63.2%, 3 = 83.3%, 4 = 71.4%, 5 = 88.9%).

At the initial visit, of patients with malodor, 68.4% of patients were treated with systemic treatment (e.g., oral metronidazole) and 8.3% received topical treatment (e.g., metronidazole gel, metronidazole spray, mupirocin ointment). For patients with bleeding, 79.3% received topical treatment (e.g., silver nitrate chemical cautery). For pain, patients were managed with topical analgesics (e.g., lidocaine ointment, morphine gel) 48% of the time, gabapentinoids 40% of the time, and opioids 24% of the time. 61.1% of patients with pruritus received topical treatment (e.g., corticosteroid ointments). All patients (n = 36, 100%) were treated with dressings (e.g., contact layer, hydrofiber, foam, superabsorbant).

### Quality of life scores

**Figure 2:**
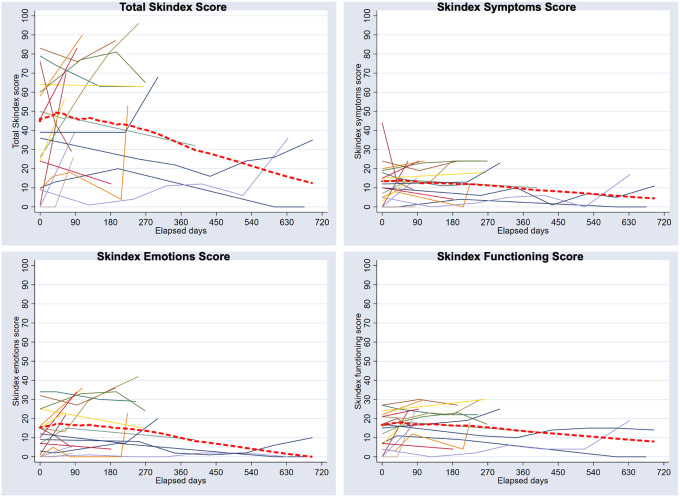
Line plot of overall Skindex 16 score, symptoms, emotions, and functioning score, for each participant over their course of treatment. The red dotted line represents the locally weighted average response for all participants.

At baseline, 36 patients completed a Skindex-16 survey, and the mean score was 54.5, categorizing their QoL as “extremely severe” (Table 3). The mean score for the symptoms, emotions, and functioning domains were 15.4, 18.8, and 20.3 respectively, with the functioning domain having the highest score. Nineteen patients completed an additional Skindex-16 questionnaire at follow-up visits (visit two 52.8%, visit three 33.3%, visit four 19.4%, visit five or greater 13.9%). Overall, patient Skindex-16 scores improved over the course of follow-up with scores decreasing, on average, 4.9 points at each interval (95% CI: 1.8–8.1, P = 0.002) (Fig. 2). The greatest decrease in Skindex-16 scores was observed between the first and second follow-up time points with scores decreasing 18.5 points (95% CI: 3.3–33.7, p = 0.018), suggesting improvement in QoL. These findings were consistent across each of the subdomains (symptoms, emotions, and functioning).

## Discussion

Malignant wounds severely reduce patients’ quality of life, and affect all QoL domains, physical, emotions, and functioning. However, patients experienced significantly improved QoL after being treated at a dedicated wound clinic. Of the patient population who continued to attend clinic and reported QoL scores, there was a decrease in physical symptoms of malodor, pain, bleeding, and pruritus with each subsequent visit. Patients with malignant wounds have a significant symptomatic burden and studies have shown that symptom control not only improves patient outcomes and physical wellbeing, but it also raises self-esteem and restores a sense of purpose (11, 12). The presence of the physical symptoms of malignant wounds such as exudate, malodor, pain, bleeding, and pruritus are strongly negatively correlated with QoL, accounting for up to 87% of variance in QoL scores in one study (6). In our study, patients with physical symptoms had a worse quality of life when compared to patients without these symptoms. The domain with the largest impact on QoL was the functioning domain, showing that malignant wounds severely impact patients’ ability to function in everyday life.

As most patients with malignant wounds have advanced cancer and are terminally ill, treatment is symptom-driven and palliative. Wound care is an important intervention in palliative care to maintain and improve QoL in this patient population. In our study, the symptom of exudate was the most common, followed by pain, bleeding, pruritus, and odor. From visit one to visit two, there was an improvement in the proportion of patients experiencing all physical symptoms. Studies have shown that using appropriate wound dressings in managing malignant wounds is critical for symptom control (13). In a cross-sectional study of 70 patients with malignant wounds in Taiwan, only 16.7% of patients were treated with dressings and only 15% had a wound specialist involved in their care (6). In comparison, most of our patients were treated with dressings (84.2%−100%) throughout wound clinic visits. However, most of our patients suffering from exudate also had a heavy degree of drainage, which may have prompted more common dressing usage.

Pain is one of the most common and distressing symptoms affecting patients with malignant wounds and is often cited as the most impactful symptom on patients’ QoL (14). In a study of the relationship between pain and malignant wound characteristics, 77.3% of patients experienced pain associated with their malignant wound, and 51.9% of patients used analgesics (15). Pain was poorly controlled, with almost half of patients not receiving adequate pain control. Our study had a similar proportion of patients receiving treatment for pain, with a majority of treatment being topical analgesics, followed by gabapentinoids, then opioids. Pain in malignant wound patients is challenging to manage and frequently chronic (14). From visit one to visit two, there was an improvement in the proportion of patients with pain. The presence of pain was shown to be a variable correlated with worse QoL scores overall and over time, which is consistent with data on the effect of pain on QoL. Patients with pruritus were commonly treated with topical corticosteroids or gabapentinoids.

Bleeding is another symptom that can affect malignant wound patients. Most patients with bleeding in our study had mild, or pinpoint bleeding. Patients with bleeding from malignant wounds have shown improvement with topical hemostatic agents such as topical silver nitrate (16), and most of our patients with bleeding were treated with topical silver nitrate chemical cautery. As with pain, the presence of bleeding also correlated with worse QoL scores in patients. Malodor is reported to be a symptom causing significant distress to patients and their families (1). Patients were most commonly treated with systemic metronidazole, followed by topical metronidazole.

Skindex-16 scores improved over the course of wound care clinic follow-up, with scores consistently decreasing at each follow-up visit. The greatest decrease in Skindex-16 scores was observed between the first and second visits, suggesting significant improvement in QoL with wound care clinic follow-up. These findings were consistent across each of the subdomains of symptoms, emotions, and functioning. This suggests that dermatologists may play a role in the palliative care team by providing wound care and alleviating the physical symptoms of malignant wounds. Overall, a multidisciplinary approach with palliative care is crucial to alleviating both the physical and psychosocial suffering of patients with malignant wounds (14, 15). However, early initiation of palliative care for cancer patients is often delayed, despite evidence for improved QoL, lower rates of depression, and increased survival rates compared to patients who receive standard care (17, 18). A study further showed that early palliative care intervention reduced cancer patients’ risk of suffering from severe pain and that patients were more likely to receive opioids (17). In our study, it is conceivable that patients who attended a wound care clinic over time had improved QoL due to the focus on palliative care and specialized management of symptoms. However, there was a significant proportion of patients who did not complete Skindex surveys at follow-up appointments. Additionally, patients who complete the Skindex surveys may not be representative of the general malignant wound population. Nevertheless, this study suggests that wound care clinics have a positive effect on patients’ symptom control and quality of life.

## Figures and Tables

**Table 1:** Characteristics of study population (n = 36 patients)

Variable		n patients (%)
Age	Mean (SD)	65.2 (16.6)
Sex	Female	25 (69.4)
	Male	11 (30.6)
Race	Asian	3 (8.3)
	Black	7 (19.4)
	Other	1 (2.8)
	Unknown	2 (5.6)
	White	23 (63.9)
Ethnicity	Hispanic/Latino	4 (11.1)
	Non-Hispanic	25 (69.4)
	Unknown	7 (19.4)
Cancer diagnosis	Breast Cancer	18 (50.0)
	Nonmelanoma skin cancer	11 (30.5)
	Sarcoma	3 (8.3)
	Non-Hodgkin lymphoma	1 (2.8)
	Prostate cancer	1 (2.8)
	Melanoma	1 (2.8)
	Thyroid cancer	1 (2.8)
Cancer stage	II	3 (8.3)
	III	15 (41.7)
	IV	18 (50.0)

**Table 2:** Clinical symptoms of study population by wound clinic visit (n = 36 patients)

	Visit #1,n = 36	Visit #2, n = 19	Visit #3, n = 12	Visit #4, n = 7
	n (%)	n (%)	n (%)	n (%)
**Odor**	17 (47.2)	4 (21.1)	2 (16.7)	1 (14.3)
**Bleeding**	23 (63.9)	5 (26.3)	5 (41.7)	2 (28.6)
**Pain**	25 (69.4)	8 (42.1)	5 (41.7)	2 (28.6)
**Pruritus**	18 (50.0)	6 (31.6)	4 (33.3)	2 (28.6)
**Exudate**	31 (86.1)	12 (63.2)	10 (83.3)	5 (71.4)

**Table 3:** Summary measures for Skindex scores at each study visit (n = 36 patients)

	Visit #1		Visit #2		Visit #3		Visit #4	
	n = 36 patients		n = 19 patients		N = 12patients		n = 7 patients	
	*Mean (SD)*	*Median (QR)*	*Mean (SD)*	*Median (QR)*	*Mean (SD)*	*Median (QR)*	*Mean (SD)*	*Median (QR)*
**Skindex 16**	54.5 (26.6)	59.5 (41)	36 (29.2)	39 (59)	42.8 (30.0)	32.5 (56)	34.4 (27.3)	16 (47)
**Skindex - Symptoms**	15.4 (8)	17.5 (13.5)	9.8 (8.6)	11 (15)	13.4 (12.1)	9.5 (13)	9.1 (7.7)	8 (17)
**Skindex - Emotions**	18.8 (13.1)	21 (21)	12.7 (12.1)	10 (24)	14.4 (13.8)	9.5 (28.5)	11.4 (13.3)	5 (24)
**Skindex - Functioning**	20.3 (8.9)	23 (13)	13.5 (10.1)	18 (18)	15 (8.2)	15 (115)	13.9 (8.2)	14 (14)
